# First Principles Study of Tritium Diffusion in Li_2_TiO_3_ Crystal with Lithium Vacancy

**DOI:** 10.3390/ma11122383

**Published:** 2018-11-27

**Authors:** Kun Li, Wen Yang, Wei-Hua Wang, Yong-Tang Li

**Affiliations:** 1 Shanxi Key Laboratory of Metal Forming Theory and Technology, School of Material Science and Engineering, Taiyuan University of Science and Technology, Taiyuan 030024, China; likun@tyust.edu.cn (K.L.); liyongtang@tyust.edu.cn (Y.-T.L.); 2 Department of Electronic Science and Engineering, and Tianjin Key Laboratory of Photo-Electronic Thin Film Device and Technology, Nankai University, Tianjin 300071, China; whwangnk@nankai.edu.cn

**Keywords:** density functional theory, tritium diffusion, lithium vacancy, Li_2_TiO_3_

## Abstract

Li_2_TiO_3_ is one of the most significant breeder materials and has potential applications in future fusion reactors. Defect models with three types of lithium vacancies were considered to study the diffusion behavior of tritium in Li_2_TiO_3_ by the density functional theory calculations. The possible tritium adsorption sites inside the lithium vacancy were examined and analyzed. The energy barrier of all diffusion paths between different adsorption sites was calculated and the minimum energy barrier is about 0.45 eV, which indicates that the tritium atom diffuses freely inside the lithium vacancy; when a tritium diffuses across the crystal in the typical three directions, our results reveal that the tritium atom prefers to move along the [010] direction. Furthermore, we found that the minimum energy barrier for the tritium atom to escape the trap of Li vacancy is 0.76 eV. After the tritium jumping out of the Li vacancy, the minimum energy barrier is 0.5 eV for the tritium atom diffusing in the crystal. Therefore, we predict that tritium can easily escape from the trap of the Li vacancy and then diffuse across the crystal. Such results are beneficial to the tritium release process in Li_2_TiO_3_ and could provide theoretical guidance for the future applications of the Li_2_TiO_3_ materials.

## 1. Introduction

Nuclear fusion is a particularly attractive clean energy source with low carbon emission in comparison with today’s conventional energy sources of coal and petroleum. Within the fusion, deuterium and tritium are fused together to form helium and a highly energetic neutron is ejected. Deuterium can be easily extracted from sea water while there is no naturally available source of tritium due to its relatively short half-life. Thus a tritium breeding blanket has been designed to continuously produce tritium (T) in the fusion reactor. In order to produce tritium, it is expedient to generate it in situ from the transmutation of lithium (Li). Lithium-containing ceramic oxides have received considerable attention as breeder materials because of their favorable properties such as high melting temperatures, low chemical reactivity, and high lithium densities [1,2]. Such lithium ceramic breeder materials will determine the power conversion efficiency and lifetime of the fusion reactor, which has become a hot topic in recent years.

A number of lithium ceramic breeder materials are being investigated both theoretically and experimentally, including Li_2_O, Li_2_TiO_3_, Li_3_TaO_4_, Li_2_ZrO_3_, Li_2_SiO_3_, and Li_4_SiO_4_ [3,4]. Among these potential tritium breeders, Li_2_TiO_3_ is considered as a candidate material because of its easy reprocessing, low activation, high lithium atom density, compatibility with structural materials, etc. [5]. So far, many efforts have been devoted to understand tritium production, accommodation, release, and related issues of lithium ceramic materials.

The current studies on Li_2_TiO_3_ mainly include the production of Li_2_TiO_3_ pebbles [6,7,8], irradiation defects and structure disorder [9,10], mechanical and thermal properties of Li_2_TiO_3_ [11,12], tritium release behavior [13,14,15,16,17], recycling process [18], and so on.

Considering the tritium release process of Li_2_TiO_3_, tritium diffusion in crystal grains has been shown to be an important step. It is affected by various irradiation-induced defects (lithium vacancy, oxygen vacancy, and broken bond). Lithium vacancy is considered to be a primary irradiation-induced defect in lithium-based breeder materials, as lithium atoms are constantly knocked out of their positions or consumed to generate tritium under irradiation.

Although several reports on tritium diffusion experiments are available in the literature, few relate to theoretical studies. Most previous theoretical studies of Li_2_TiO_3_ focus on the structural, electronic, and dynamical properties of the perfect crystal [5,12]. Concerning the defect model, Murphy studied tritium adsorption in the Li vacancy of Li_2_TiO_3_ [19] and Islam et al. studied Li diffusion in Li_2_TiO_3_ [20]. Moreover, due to the important role of lithium vacancy in tritium release, we believe it is necessary to study the impact of vacancy working on tritium diffusion behavior in Li_2_TiO_3_. To the best of our knowledge, there has not been any reported theoretical work on tritium diffusion behavior with vacancy in Li_2_TiO_3_ so far, yet there is no clear explanation about the tritium diffusion process in Li_2_TiO_3_.

In this work, on the atomistic scale, a comprehensive computational study of tritium diffusion inside lithium vacancy and the tritium diffusion across the crystal of Li_2_TiO_3_ are presented. Diffusion pathway, energy barrier, and electronic structure of tritium with all types of lithium vacancies in Li_2_TiO_3_ are calculated and analyzed systematically. These theoretical calculations of tritium diffusion in lithium vacancy of Li_2_TiO_3_ are of prime importance for the understanding of tritium behavior in Li_2_TiO_3_ and also design of the tritium breeding blanket.

## 2. Theoretical Model and Methods

The calculations have been performed by using the density functional theory (DFT) and the plane wave method, within the Vienna ab initio simulation package (VASP) [21,22]. The projector-augmented wave method (PAW) [23] was used to describe the core ion and valence electron interaction. The generalized gradient approximation (GGA) of Perdew–Burke–Ernzerhof (PBE) [24] was used; the energy barriers in the considered pathways were treated with the climbing image nudged elastic band (NEB) scheme [25,26,27].

Convergence tests were conducted to determine the energy cutoff of the plane wave basis and the K-point grid. Finally the plane wave cutoff energy was set as 500 eV, and we performed geometry optimization with 2 × 2 × 2 Monkhorst–Pack grids K-point and 6 × 6 × 6 for electronic structure calculation. The Gaussian band smearing method with a width of 0.05 eV was utilized. The electronic self-consistent loops were converged to 10^−4^ eV/cell and the geometry relaxation was considered to be completed when the total force on the atom is less than 0.003 eV/Å. Both the atom positions and the size/shape of the cell were allowed to change during the geometry relaxation. All tritium atoms were treated as hydrogen atoms as they present the same electronic properties within DFT calculations.

## 3. Results and Discussion

Here defect models of Li_2_TiO_3_ with three types of Li vacancies are set up and simulated by density functional theory. The adsorption and diffusion of a tritium atom in the Li vacancies and across the defect crystal have been thoroughly investigated and analyzed.

### 3.1. Formation Energy of Lithium Vacancy

**Figure 1:**
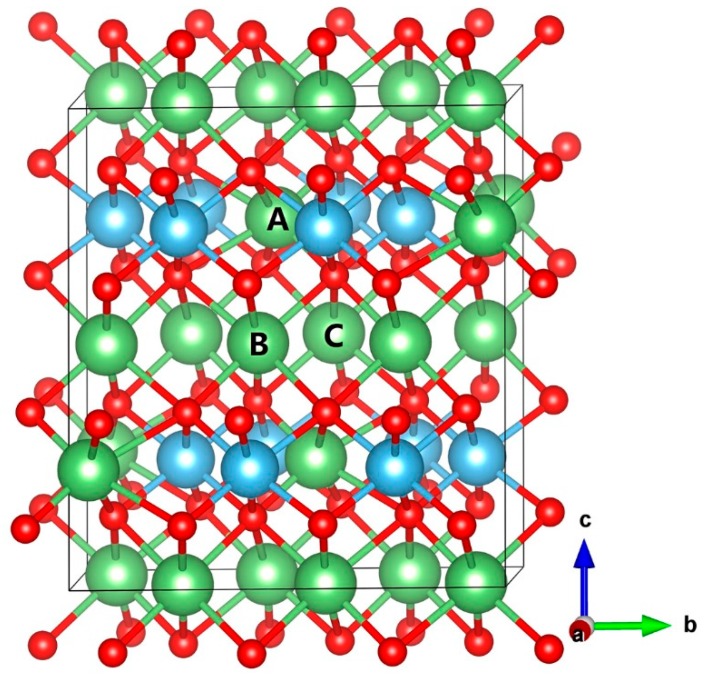
The structure of the β-Li_2_TiO_3_ crystal (color online). Lithium atoms are represented by green balls, titanium atoms are blue balls, and oxygen atoms are red balls. The labels of A, B, and C correspond to the three lithium atom positions, respectively.

β-phase Li_2_TiO_3_ was considered a stable phase until 1215 °C, and its crystal structure was first determined by Lang [28] and subsequently refined using X-ray diffraction of large single crystals by Kataoka et al. [29]. The β-Li_2_TiO_3_ crystal consists of alternating LiTi_2_ layers and pure Li layers. The experimental lattice parameters of β-Li_2_TiO_3_ are a = 5.062 Å, b = 8.788 Å, c = 9.753 Å, and β = 100.212° with the space group C2/c [29]. There are 48 atoms in each cell in total, of which 16 there are lithium, eight titanium and 24 oxygen atoms. The detailed structure data including the atom coordinate and Wyckoff site are presented in Table 1. According to Table 1, there are three types of crystallographically inequivalent Li positions, namely, Li type A, Li type B, and Li type C, as shown in Figure 1.

In our study, according to the above experimental parameters, a 2 × 1 × 1 supercell of Li_2_TiO_3_ was employed and then its geometry was optimized using DFT calculations. The defect formation energy of a lithium vacancy *E_f_*(*V_Li_*) is then given by

(1) $$E_{f}(V_{Li})=E(Li_{2}TiO_{3}:V_{Li})-E(Li_{2}TiO_{3})+E(Li_{metal}),$$

where *E*(*Li*_2_*TiO*_3_:*V_Li_*) is the total energy of the defect model, *E*(*Li*_2_*TiO*_3_) is the total energy of the 2 × 1 × 1 super cell of the perfect crystal, and *E*(*Li_metal_*) is the per atom energy of the bcc Li metal crystal.

**Figure 2:**
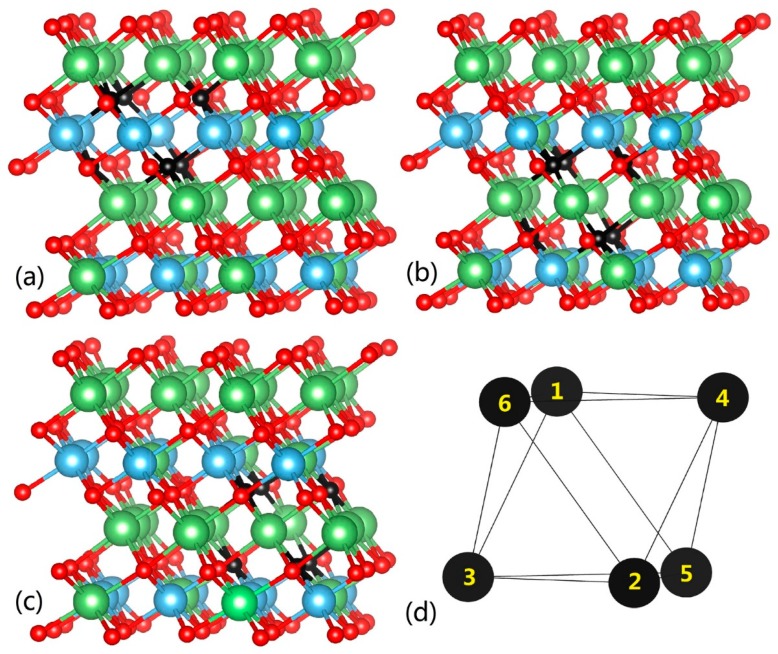
The defect model of Li_2_TiO_3_ with three types of Li vacancies (color online). (**a**) Type A, (**b**) Type B, (**c**) Type C, and (**d**) sketch map of the octahedron configuration of each type vacancy. The black balls represent oxygen atoms.

Initially, we relax the perfect crystal Li_2_TiO_3_ model and obtain the lattice parameters of β-Li_2_TiO_3_ as a = 5.129 Å, b = 8.904 Å, c = 9.851 Å, and β = 100.213°, which is very close to the experimental results [29] and previous simulations [19,20]. Furthermore, systematic study was performed on all the three types (type A/B/C) of lithium vacancies in Li_2_TiO_3_. By removing one type of lithium atom from the perfect crystal, three defect models of Li_2_TiO_3_ were created and the corresponding lithium vacancy structures were plotted in Figure 2a–c.

Each lithium vacancy constitutes an octahedral configuration with 6 oxygen atoms at vertex positions in Figure 2d and lithium atom in the center. These models were subsequently relaxed and afterwards the formation energy of the three types of lithium vacancies were obtained, which corresponded to 2.940 eV for type A, 3.029 eV for type B, and 3.029 eV for type C. The difference between the highest and the lowest formation energy is about 0.09 eV, which is close to the 0.12 eV obtained by Murphy et al. [19].

### 3.2. Tritium Adsorption in Lithium Vacancy

The adsorption of tritium in different Li vacancies was investigated firstly because the diffusion of a tritium atom takes place between different stable and meta-stable sites. The adsorption energy of tritium trapped in lithium vacancy *E_f_*(*T*) can be written as

(2) $$E_{f}(T)=E(Li_{2}TiO_{3}:T)-E_{f}(Li_{2}TiO_{3}:V_{Li})-\frac{1}{2}E(T_{2}),$$

where *E*(*Li*_2_*TiO*_3_:*T*) is the energy of the Li_2_TiO_3_ system containing both a lithium vacancy and a tritium atom, and $\frac{1}{2}E(T_{2})$ is the atom energy of tritium.

Here we calculated the adsorption energy of a tritium in three types of vacancies (type A/B/C), respectively. In such vacancies, we found that the oxygen dangling bonds created by the removal of lithium atom would be the most probable sites for tritium atom. Thus there are six adsorption sites for the tritium atom in each vacancy. As was shown in Figure 2d, sites labeled as site 1 to site 6 constitute an octahedral configuration. The adsorption energy of a tritium in six sites of each type Li vacancy was presented in Table 2. For instance, A-site 1 represents the results for the tritium adsorption at the position of site 1 in the Li_2_TiO_3_ crystal with type A Li vacancy. In all the cases, the obtained tritium–oxygen (T–O) bond length is around 0.98 Å with very small deviation of no more than 0.01 Å, which is very close to the 0.985 Å predicted by Murphy [19] and is slightly shorter than the 1.02 Å predicted by Shein et al. [30]. All the T–O bonds point toward to the approximate center of vacancy, and no distinct differences are observed for the T–O bond.

In the vacancy of type A, the adsorption energy of six sites are very close to each other (−4.6 eV in Table 2) and the difference is less than 0.02 eV. While the corresponding cases in the vacancies of type B and type C are exactly the same due to the C2/c symmetry properties. Thus the type B and type C vacancies are equivalent and the type C vacancy will not be specifically discussed in the following. Moreover, in the type B vacancy, the maximum adsorption energy is approximately −4.49 eV at site 1 and site 3, and the minimum is approximately equal to −4.71 eV at site 2 and site 4. The difference between maximum and minimum adsorption energy in the vacancy of type B is approximately 0.22 eV which is much larger than that in the vacancy of type A.

To explain this energy difference, we calculated the mean bond length between oxygen and its neighboring three lithium atoms (mean O–Li bond length) with a tritium trapped in the vacancy B. The obtained mean O–Li bond length is about 2.25 Å at B-site 1/site 3 and is approximately 2.19 Å at B-site 2/site 4. The latter is closer to the average O–Li bond length of 2.15 Å in the perfect Li_2_TiO_3_ crystal. Hence we infer the fact that the bonding energy decreases as the O–Li bond length extends which leads to the increasing adsorption energy of tritium at the site 1/site 3.

The obtained tritium adsorption energy (−4.49–−4.71 eV) indicates the strong adsorption of the tritium atom in the Li vacancy. And the results predict the lithium vacancy in Li_2_TiO_3_ has different tritium trapping capabilities. Analysis in-depth reveals that such capabilities are influenced by the O–Li structure around the vacancy.

### 3.3. Tritium Diffusion Inside the Lithium Vacancy

We used NEB calculations to probe the tritium diffusion in the lithium vacancy of the bulk Li_2_TiO_3_ in this section. Energy barriers for all the vacancy-assisted transition paths found in this study were summarized in Table 3. Of all the fifteen paths, there are twelve paths called “edge path” (paths 1–12 in Table 3) which are pathways along the edge line of the octahedral vacancy. For example, path 1 is from site 1–4 while its direction is in the edge line as illustrated in Figure 2d. Path a/b/c in Table 3 are “diagonal paths” along the diagonal line of the octahedron, as is pathway a from site 1–2, as shown in Figure 2d. The results indicate that in all the octahedral vacancies, the energy barrier of diffusion along the diagonal path is higher than that along the edge path. Such fact means that the tritium prefers to move along the edge of the octahedron inside the vacancy.

In the vacancy of type A, as shown in Table 3, the maximum energy barrier of the edge path is 0.86 eV in path 4, and the minimum is 0.63 eV in path 9. Meanwhile the energy barrier of the three diagonal paths (path a, path b, and path c) are 2.97, 2.97, and 2.98 eV, respectively, which is much higher than that of the edge paths. The maximum energy barrier of the edge path in type B vacancy is 0.98 eV in path 12, and the minimum is 0.45 eV in path 5; the energy barriers of the three diagonal paths are correspondingly 3.07, 3.11, and 3.00 eV.

The energy barrier difference in different paths is probably related to the length of the paths. The corresponding lengths for all the paths are illustrated in Table 3 as well. It was found that the energy barrier is generally high when the path length is long and is opposite when the length is short. For example, the longest edge paths in the type B vacancy are path 6 and path 12 with path lengths of 1.99 Å, which correspond to the highest energy barrier of the edge paths. On the contrary, the path length is only 1.34 Å of path 5, and the energy barrier is the lowest 0.45 eV of all the fifteen paths.

**Figure 3:**
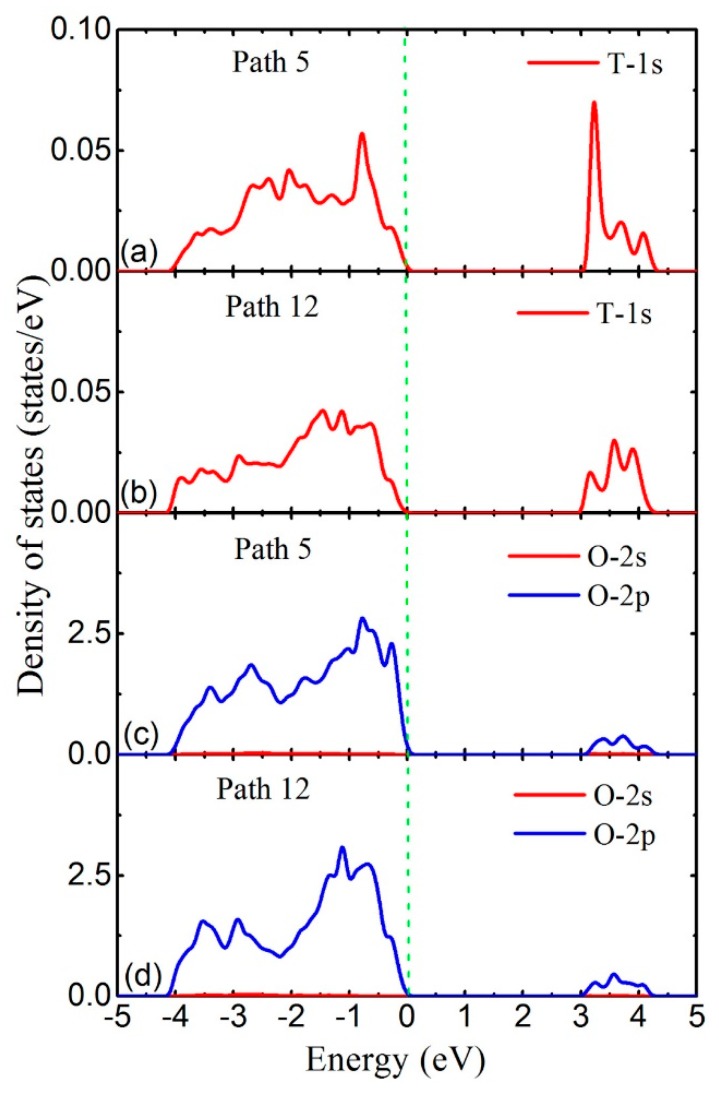
(**a**,**b**) The LDOS of tritium (T) atom with the highest energy barrier in the diffusion path 5 and path 12 (color online). (**c**,**d**) The LDOS of the oxygen (O) atom at both ends of diffusion path 5 and path 12. The green vertical dotted line is the Fermi level which was set to zero.

Moreover, we analyze the local density of states (LDOS) to deeply explain the energy barrier difference for distinct paths. For example, the LDOS of T atom in edge paths for the lowest energy barrier (path 5) and highest energy barrier (path 12) in crystals with type B vacancy, plotted in Figure 3a,b, respectively. The LDOS of oxygen (O) atoms at both ends of path 5 and path 12 are presented in Figure 3c,d, respectively. A broad peak exists when the electron energy is below Fermi energy to −4 eV in Figure 3a–d. Such phenomena means that the 1s electron of the tritium atom bonds with the 2p electrons of oxygen in both paths 5 and 12. Furthermore, the electron population of the tritium–oxygen (T–O) bonds is calculated and the results are 0.93 for path 5 and 0.87 for path 12. This fact indicates that the T–O interaction is stronger during tritium diffusing in path 5 which leads to the lower energy barrier of the diffusion.

Hence we infer that the T–O interaction plays an important role in determining the energy barrier of the diffusion path. Accompanied with the increasing length of the diffusion path, the distance between tritium atom and oxygen atom is getting longer which may lead to the bond breaking and the weakened T–O interaction, and eventually leading to the high diffusion energy barrier.

It is worth noting that the diffusion path with a high energy barrier actually could be implemented by the combination of other paths with lower energy barrier. For instance, path 12 of the type B vacancy is diffusing from site 5 to site 4 (Table 3). This diffusion path may be achieved by diffusion first from site 5 to site 2 (path 8) and then from site 2 to site 4 (path 5), which reduces the energy barrier from site 5 to site 4 to 0.62 eV. From this point of view, the maximum energy barrier is 0.74 eV for the tritium atom diffusion from one stable adsorption site to another site in the type B vacancy; the energy barrier inside the type A lithium vacancy is less than 0.85 eV.

It could be concluded from the above results that the energy barrier for a tritium atom needs to overcome to diffuse from one stable site to another is no more than 0.9 eV (the minimum is 0.45 eV). This means that the tritium atom diffuses easily between different stable sites in the vacancy at low temperature, which provides possibilities of the tritium to escape from the vacancy.

### 3.4. Tritium Diffusion Across the Crystal

In this section, the diffusion of a tritium atom across the crystal is studied. The main target is to understand how the tritium atom escapes from the vacancy and diffuses in the crystal. Preliminary tests show that the tritium atom is always adsorbed to the oxygen atom after the geometry optimization regardless of the initial position. Thus the diffusion of tritium across the crystal mainly corresponds to the diffusion through different oxygen adsorption sites. As the Li_2_TiO_3_ crystal consists of four types of oxygen atoms and each type has several metastable adsorption sites, there are huge numbers of diffusion paths in the crystal, and it is convenient for us to choose typical paths to calculate due to the current limitations of computational capabilities.

Three typical directions of [100], [010], and [001] are considered to study the tritium diffusion pathway across the crystal according to the symmetry. As preliminary tests have shown that the tritium atom is always adsorbed to the oxygen atom, it is natural to choose the tritium adsorption sites on the oxygen atoms as the nodes to connect the diffusion pathway. At the same time, the pathway should go across the Li vacancy and the shortest diffusion pathway is favored because the above results have proved that the diffusion energy barrier is lower for the shorter pathway. Considering all of the above factors, the diffusion pathway in each direction is finally determined after huge number of tests.

**Figure 4:**
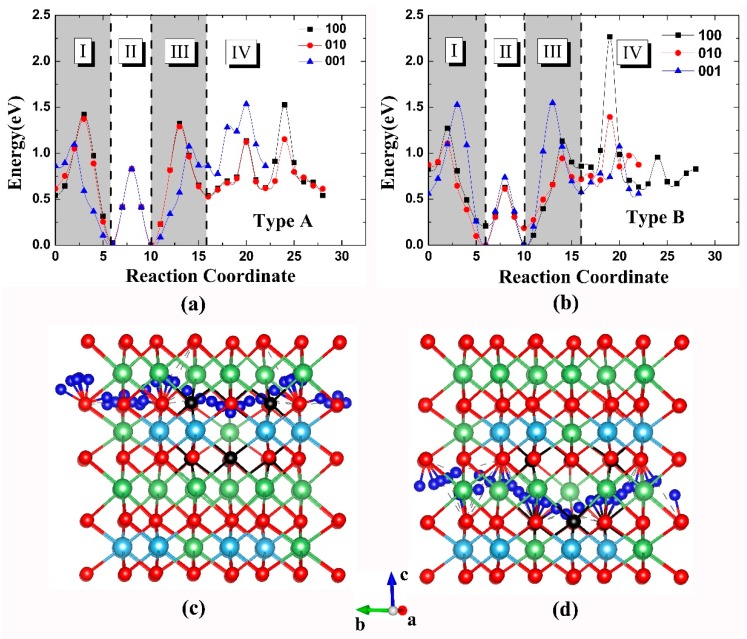
(**a**,**b**) The energy barrier of three tritium diffusion paths with the direction of [100], [010], and [001] in the crystal with the vacancy of type A or B (color online). Parts I–IV are separated by vertical dashed lines. (**c**,**d**) The diffusion pathway along the [010] direction in the crystal with the vacancy of type A or B.

For the crystal with the vacancy of type A or B, the energy barrier of three diffusion paths corresponding to the direction of [100], [010], and [001] were calculated and presented in Figure 4a,b. In Figure 4a, the overall minimum energy barrier is 1.37 eV for the whole path in the [010] direction. The diffusion pathway profile for the tritium atom along this direction was plotted in Figure 4c. In the case of type B vacancy, the minimum energy barrier is 1.39 eV, which is also found for the [010] direction; the diffusion path profile was drawn in Figure 4d. Such results imply that the tritium atom prefers to move along the [010] direction when diffusing across the crystal.

Further exploring the diffusion process of the tritium atom in the bulk Li_2_TiO_3_, we find that each diffusion path can be divided into four parts (I–IV) as illustrated in Figure 4a,b. For part I, it corresponds to the tritium diffusion from the crystal to the Li vacancy, namely the tritium diffuses from the adsorption site from a saturated oxygen atom to another site of an oxygen atom with dangling bond. The part II is the tritium diffusion inside the Li vacancy which has been fully discussed in the last section. The part III is the tritium diffusion away from Li vacancy to crystal, in a direction opposite to that of part I. The part IV is the tritium diffusion in the crystal after jumping out of the Li vacancy which corresponds to the diffusion through different saturated oxygen atoms in the Li_2_TiO_3_ crystal. These four parts constitute a completed diffusion model including “diffusion-trapping-detrapping-diffusion” processes.

The energy barrier for each part is presented in Table 4. It is found that the minimum energy barrier for the tritium escaping from the Li vacancy is 0.76 eV, which corresponds to the diffusion of part III in the [010] direction in the crystal with type B Li vacancy. While in the case of part IV diffusion, the lowest energy barrier is 0.50 eV when the tritium diffuses along [001] direction in the crystal with type B Li vacancy. For the part II diffusion, the minimum energy barrier in Table 4 is 0.63 eV which is higher than 0.45 eV obtained in the last section. That is because here we only calculate the diffusion paths in three directions. Moreover, the “null” in Table 4, such as the “null” in [100] direction, implies that there is no part II diffusion in the pathway, namely the tritium does not diffuse inside the trap of the Li vacancy when diffuse across the crystal in the [100] direction. Comparing the four diffusion parts, the minimum energy barrier for each part is ranging from 0.45 to 0.76 eV; these results are consistent with the previous experiments which will be analyzed in the following.

Several experiments have been applied to estimate the activation energies of the tritium diffusion in the neutron-irradiated solid breeding materials, including thermal desorption spectroscopy (TDS), isothermal heating, and so on [31,32,33,34,35,36,37,38]; the activation energies predicted by related experiments normally range from 0.59 to 1.55 eV [31,32,33,34]. Thus we conclude that the scope of our calculated minimum energy barrier is consistent with the experiments. And our diffusion model clearly expresses the diffusion process of the tritium across the Li_2_TiO_3_ crystal with Li vacancy.

**Figure 5:**
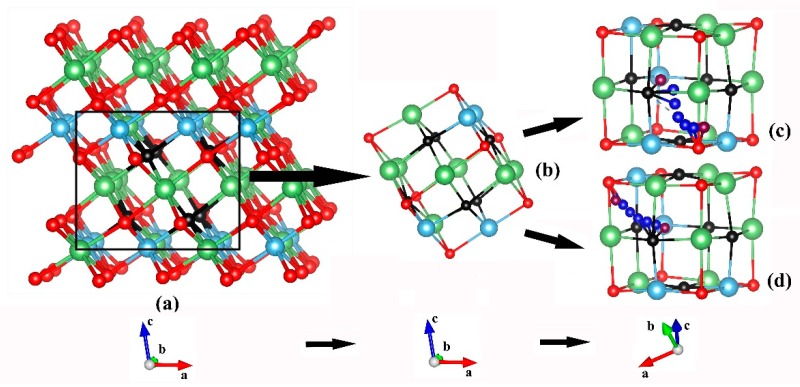
(**a**) shows the relative position in the crystal of the vacancy structure of (**b**) (color online). (**c**,**d**) The diffusion profile of part III diffusion away from the type B Li vacancy to the crystal in directions of [001] and [010], respectively.

Besides, it has been found that the initial and ending tritium positions are of major importance to the diffusion energy barrier. The part III diffusion profiles for tritium escaping from the type B Li vacancy to the crystal in directions of [001] and [010] are plotted in Figure 5c,d, respectively. Figure 5a,b show the relative location of the vacancy in the bulk crystal. In the vacancy of Figure 5c,d, the purple ball bonding with black oxygen atom (inside vacancy) is the initial tritium position and the purple ball bonding with red oxygen atom (outside vacancy) is the ending tritium position. The length and minimum energy barrier of the part III diffusion path are 4.43 Å, 1.51 eV in [001] direction, and 2.95 Å, 0.76 eV in [010] direction. Figure 5c shows that a Li atom is locating quite close to the diffusion path, which leading to the extra diffusion path length and energy barrier because the tritium has to diffuse away from the Li atom. This explains why the energy barrier in the [001] direction is much higher than that of the [010] direction. Such phenomenon also happens in other diffusion process. For example, the high peak in the Figure 4b when tritium diffuses in part IV in the [100] direction may be due to this fact.

Generally, a completed diffusion model including “diffusion-trapping-detrapping-diffusion” processes is set up to explain the tritium diffusion behavior in the Li_2_TiO_3_ crystal with Li vacancy. Typical diffusion paths in three directions were calculated and analyzed in four parts. The obtained minimum energy barrier is less than 0.76 eV which show that the tritium is easy to escape from the trap of the Li vacancy and diffuse across the crystal.

## 4. Conclusions

In this work, a systematic study of the diffusion behavior of a tritium atom inside all three types of lithium vacancies in Li_2_TiO_3_ and across the defect complexes were investigated using the first principles method. Based on the calculated formation energies, adsorption energies, and electronic structures of the tritium trapped system, possible tritium adsorption sites inside the lithium vacancy were studied in detail and adsorption sites with minimum adsorption energy were determined firstly. With further investigation we reveal that the trapping capabilities for the different adsorption sites are influenced by the O–Li interaction around the lithium vacancy.

Then a completed diffusion model including “diffusion-trapping-detrapping-diffusion” processes is set up to explain the tritium diffusion behavior inside the Li vacancy and across the bulk Li_2_TiO_3_. The obtained minimum energy barrier for the tritium diffusing inside the Li vacancy is 0.45 eV; the minimum energy barrier is 0.76 eV for tritium escaping out of the trap of the Li vacancy. Then the minimum energy barrier is 0.50 eV when tritium diffusing across the crystal after jumping out of the Li vacancy. Due to our limited calculations of the diffusion paths, it is possible that there is even lower energy barrier for the tritium diffusion in the bulk Li_2_TiO_3_ with Li vacancy. Therefore we predict that it is easy for tritium to escape from the trap of the Li vacancy and diffuse across the crystal; our diffusion model gives a clear expression of the tritium diffusion behavior which could further help to understand the experiments. Our results shed light on the tritium release process and provide further support of the Li_2_TiO_3_ as the breeder materials.

## Figures and Tables

**Table 1:** Structure data of Li_2_TiO_3_ crystal.

Atom Type	Fractional Coordinates of Atoms (x,y,z)	Wyckoff Sites
Li type A	0, 0.045, 0.250	4e
Li type B	0.238, 0.077, 0	8f
Li type C	0.250, 0.250, 0.500	4d
Ti type A	0, 0.415, 0.250	4e
Ti type B	0, 0.747, 0.250	4e
O type A	0.141, 0.265, 0.138	8f
O type B	0.102, 0.586, 0.138	8f
O type C	0.138, 0.906, 0.135	8f

**Table 2:** Adsorption energy of tritium atom in different sites of three types of Li vacancies.

Vacancy Type-Site	Adsorption Energy (eV)	T-O Bond Length (Å)
A-Site 1	−4.59	0.99
A-Site 2	−4.61	0.99
A-Site 3	−4.60	0.99
A-Site 4	−4.60	0.99
A-Site 5	−4.59	0.99
A-Site 6	−4.61	0.99
B-Site 1	−4.49	0.98
B-Site 2	−4.71	0.99
B-Site 3	−4.49	0.98
B-Site 4	−4.70	0.99
B-Site 5	−4.53	0.98
B-Site 6	−4.53	0.98
C-Site 1	−4.49	0.98
C-Site 2	−4.71	0.99
C-Site 3	−4.49	0.98
C-Site 4	−4.70	0.99
C-Site 5	−4.53	0.98
C-Site 6	−4.53	0.98

**Table 3:** Energy barrier for different tritium diffusion paths in type A and type B vacancies.

	Pathway	Type A		Type B	
		Diffusion Barrier (eV)	Length (Å)	Diffusion Barrier (eV)	Length (Å)
1	Site 1–4	0.64	1.41	0.96	1.93
2	Site 1–6	0.75	1.44	0.63	1.39
3	Site 1–3	0.85	1.85	0.71	1.62
4	Site 1–5	0.86	1.86	0.80	1.86
5	Site 2–4	0.83	1.84	0.45	1.34
6	Site 2–6	0.82	1.83	0.97	1.99
7	Site 2–3	0.66	1.40	0.95	1.92
8	Site 2–5	0.75	1.44	0.62	1.61
9	Site 4–6	0.63	1.40	0.61	1.63
10	Site 6–3	0.83	1.84	0.74	1.85
11	Site 3–5	0.64	1.41	0.62	1.62
12	Site 5–4	0.85	1.85	0.98	1.99
a	Site 1-2	2.97	2.27	3.07	2.28
b	Site 3-4	2.97	2.25	3.11	2.29
c	Site 5-6	2.98	2.27	3.00	2.58

**Table 4:** Energy barrier for diffusion parts I–IV of diffusion paths in three directions.

	Energy Barrier (eV)		
	[100]	[010]	[001]
A-Part I	1.39	1.37	1.10
A-Part II	null	null	0.83
A-Part III	1.32	1.29	1.10
A-Part IV	0.98	0.59	0.67
B-Part I	1.27	1.10	1.53
B-Part II	0.63	0.63	0.75
B-Part III	1.13	0.76	1.51
B-Part IV	1.63	0.68	0.50
